# (Sub)structure Development in Gradually Swaged Electroconductive Bars

**DOI:** 10.3390/ma16155324

**Published:** 2023-07-28

**Authors:** Jaromír Kopeček, Lucia Bajtošová, Petr Veřtát, Daniel Šimek

**Affiliations:** 1 FZU—Institute of Physics of the Czech Academy of Sciences, Na Slovance 2, 18200 Prague, Czech Republic; vertat@fzu.cz (P.V.); simek@fzu.cz (D.Š.); 2 Department of Physics of Materials, Charles University in Prague, Ke Karlovu 5, 12116 Prague, Czech Republic; lucibajtos@gmail.com

**Keywords:** rotary swaging, electrical conductivity, copper, microstructure, texture, EBSD

## Abstract

Copper generally exhibits high electrical conductivity but has poor mechanical properties. Although alloying can improve the latter characteristic, it usually leads to a decrease in electrical conductivity. To address this issue, a promising approach is to enhance the performance of copper while maintaining high electrical conductivity through optimized deformation processing, which refines the structure and increases mechanical properties. This paper focuses on assessing the effects of rotary swaging, a form of deformation processing, on microstructures and substructures of electroconductive copper bars. This analysis is complemented by experimental measurements of electrical conductivity. The results demonstrate that gradual swaging, i.e., applying different swaging ratios, influences the structure-forming processes and consequently affects the electrical conductivity. The increased electrical conductivity was found to be associated with the elongation of the grains in the direction of the electron movement.

## 1. Introduction

The microstructure, which encompasses the overall properties of grains and includes their morphology, size, and type of boundaries, plays a crucial role in determining the behaviors and properties of a given metallic material [1]. Currently, the majority of metallic materials are prepared via either methods based on bulk casting [2,3,4] or techniques based on the processing of metallic powders (e.g., mechanical alloying [5,6], cold and hot sintering [7,8], selective laser sintering (SLS) [9,10], powder bed fusion (PBF) methods [11,12], and more). In order to enhance the microstructures of cast/sintered workpieces, deformation processing can be employed as an additional production step. Moreover, plastic deformation methods can also be utilized to modify the geometry of the prepared workpiece or to impart the final shape to the product. Conventional plastic deformation processes include (die) forging [13,14], drawing [15,16], rolling [17,18], extrusion [19,20], and others. Alternatively, unconventional methods such as severe plastic deformation (SPD) [21,22,23,24,25,26], radial forging [27,28], and rotary swaging [29,30] can also be employed.

During the unconventional (severe and intensive) methods of plastic deformation, the processed workpieces are subjected to severe or intensive shear strain, which primarily introduces grain refinement through the simultaneous occurrences of grain fragmentation and substructure development [31]. In other words, the grains repeatedly fragment, resulting in the nucleation of dislocation substructures and subgrains (i.e., development of low-angle grain boundaries, LAGB), eventually leading to the development of fully refined grains (characterized by high-angle grain boundaries, HAGB). This leads to the enhancement of (mechanical) properties (e.g., strengthening) of the processed metallic materials [32,33]. However, the rate and extent of the development of these structural features are primarily affected by the processing conditions and the imposed strain, i.e., the deformation ratio.

Rotary swaging is a method of intensive plastic deformation that can be advantageously used to simultaneously enhance the properties of the processed material by imparting (repeated) grain fragmentation and restoration while achieving the desired geometry of the product. Swaging can be applied under hot [34], warm [35], and cold [36] conditions, either as a sole processing method [37] or in combination with another (unconventional) plastic deformation method (e.g., SPD methods [38,39]).

Because of its incremental character and favorable stress state during processing [40], this method is suitable also for the processing of materials with low plasticity (e.g., Mg-based alloys [41,42] or hardenable Al-based alloys [43,44]), various composites [45,46], or gradient materials [47,48]. It can even be used for postprocessing powder-based materials to enhance compactness, eliminate voids, increase mechanical properties, and extend their lifetime [49,50]. However, regardless of the processed material, rotary swaging not only significantly influences the grain size of the final product but also can affect the grain orientations, i.e., texture; this has a non-negligible influence not only on the (possible anisotropy of) mechanical properties but also on other factors like electrical properties.

Cu is known for its excellent electrical conductivity, and the worldwide standard for assessing the electrical conductivity of materials (the International Annealed Copper Standard, IACS) is derived from the properties of this metal. Its electrical conductivity, however, depends on its purity, with the highest-purity Cu exhibiting the highest electrical conductivity achieved through electrolytic refining methods, which eliminate any impurities within the structure [51]. Nonetheless, pure Cu suffers from poor mechanical properties, which can be improved by adding alloying elements. This provides strengthening via formation of precipitates and secondary phases, as well as interstitial strengthening [52,53]. On the other hand, the addition of such elements also results in the deterioration of electrical conductivity [54,55]. Nevertheless, optimized deformation processing can be advantageously employed to ensure both increased mechanical properties and, simultaneously, high electrical conductivity. The primary aim of the study presented herein is to investigate the (sub)structure within the electroconductive Cu bars produced by cold rotary swaging. The focus is on the correlation of the observed (sub)structural phenomena with the selected processing conditions, i.e., the applied reduction ratio. To explore the effects of deformation processing on the behavior of the electroconductive swaged bars, this study is supplemented with experimental measurements of the electrical conductivity.

## 2. Materials and Methods

The original material used for the experiments was commercially available, commercially pure (CP) electroconductive Cu (plus 0.016 wt.% P, 0.002 wt.% Zn, and 0.001 wt.% O). The composition and homogeneity of the processed material was verified by EDS analysis during the microstructure investigation. Before commencing the experimental work, the material underwent heat treatment at 600 °C for 30 min to homogenize and relax the structure. Subsequently, the original material was cut into billets with an initial diameter of 50 mm, which were then subjected to gradual rotary swaging at room temperature. The swaging process gradually reduced the diameters of the bars to 20 mm, 15 mm, and 10 mm. The reduction ratio after each swaging pass was calculated using Formula (1),

(1) $$\phi =\ln\left(\frac{S_{0}}{S_{n}}\right)$$

where *S*_0_ and *S_n_* are the areas of cross-sections of the bar at the input and output of the dies, respectively. The calculated values of reduction ratios for the individual swaging passes are summarized in Table 1.

**Figure 1:**
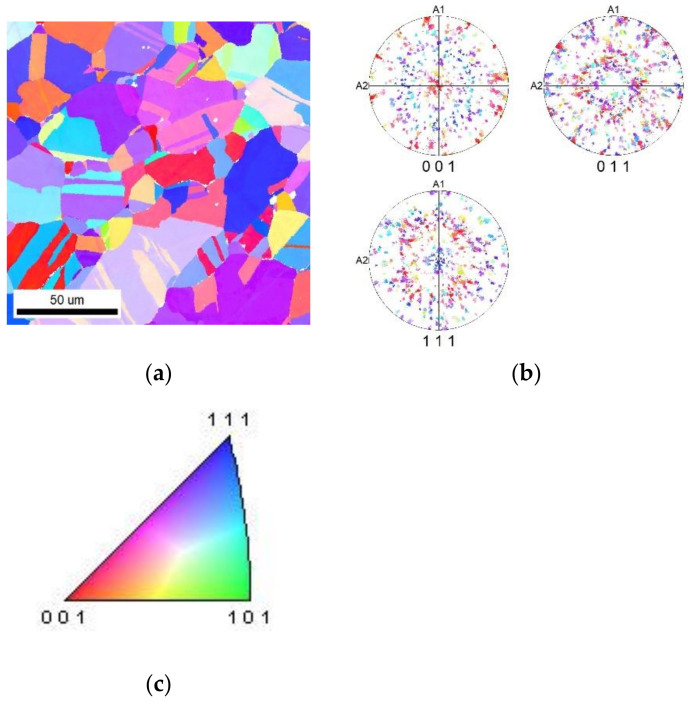
Orientation map (OIM) image for original commercial purity Cu (**a**) and its pole figure (PF) (**b**). Grains are isotropic with grown twins due to homogenization during annealing. The pole figure does not show texture. The color code for OIM maps is given in part (**c**).

After swaging, samples from each swaged bar were subjected to scanning electron microscopy (SEM) using a Tescan FERA 3 device (Tescan Orsay Holding a.s., Brno, Czech Republic). The composition was examined using an energy dispersive spectroscopy (EDS) EDAX Octane Super 60 mm^2^ analyzer, and the orientation of the crystal lattice was examined by an electron back-scattered diffraction method (EBSD) EDAX DigiView V camera. Data acquisition was performed in APEX 2.5 software, and EBSD maps were evaluated using OIM 8.6 software (all EDAX, Ametek Inc., Berwyn, PA, USA). As the imposed strain was expected to be the highest at the periphery of the swaged bars [56,57], samples from the peripheral locations of the bars were examined. The samples were cut by electrical discharge machine (EDM; ZAP BP 05dW, Kutno, Poland) from bars and mounted into epoxy resin to avoid heating. The samples were mechanically polished using a contemporary procedure for copper and subsequently electrochemically polished with D2 electrolyte. To evaluate the initial state of the CP Cu, a sample of the original material was also subjected to SEM examination. Figure 1a depicts the orientation image map (OIM) of the original Cu after the applied heat treatment, and Figure 1b shows the (001), (011), and (111) pole figures (PF) of the structure, documenting the more or less random orientations of the individual grains in the stereographic projection.

Detailed substructure analyses and observations were carried out using transmission electron microscopy (TEM) on a JEOL 2000 FX (JEOL Inc., Akishima, Japan). The samples were prepared electrochemically.

To examine the influence of the swaging procedure on the electrical behavior of the bars, the electrical conductivity of the processed conductors was assessed using SIGMATEST 2.070 equipment (FOERSTER TECOM s.r.o, Prague, Czech Republic). This portable device for measuring the electric properties of non-ferromagnetic specimens is based on eddy currents and contains a high-tech measuring probe whose function is based on complex impedance. Before conducting the experimental measurements, calibration of the device was performed using two calibration specimens with known electrical conductivity. Subsequently, measurements of the swaged rods were performed.

## 3. Results

### 3.1. Grain Size

**Figure 2:**
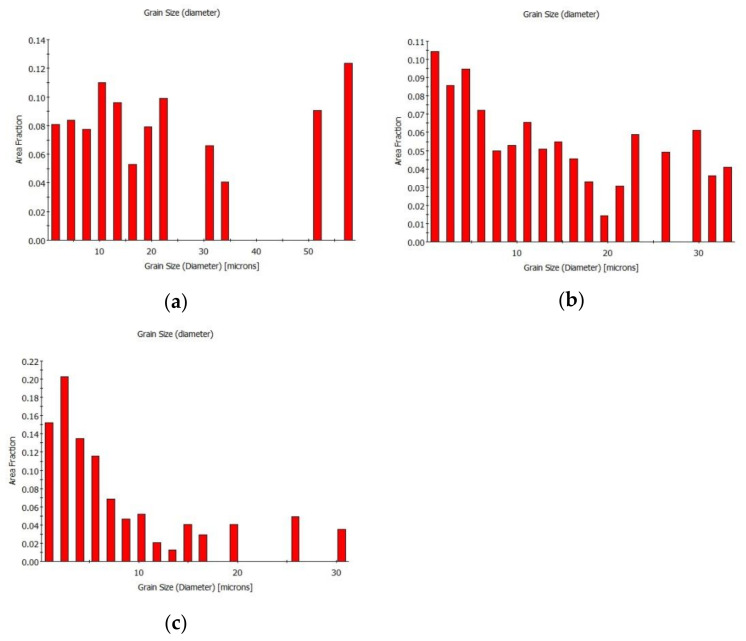
Grain size charts for swaged Cu samples: *bar20* (**a**), *bar15* (**b**), and *bar10* (**c**).

The initial focus of the structure analyses was to assess the grain size within the individual swaged bars. Figure 2a–c present the grain size charts obtained from cross-sectional scans for samples from the swaged rods, namely, *bar20*, *bar15*, and *bar10*. Figure 2a reveals a bimodal grain distribution in the structure of the *bar20* sample, indicating the presence of small grains with diameters smaller than 10 µm and large grains with diameters of about 50 µm. Swaging to the diameter of 15 mm led to grain size homogenization and also a decrease in the average grain size. Figure 2b displays the grain size chart for the *bar15* sample, demonstrating more or less uniform distribution of grain sizes through the observed size range. The final swaging pass to the diameter of 10 mm (*bar10* sample) led to a significant grain refinement. The fraction of grain sizes with diameters smaller than 5 µm increased substantially, covering approximately 50% of the total area fraction (Figure 2c).

### 3.2. Orientations of Grains

**Figure 3:**
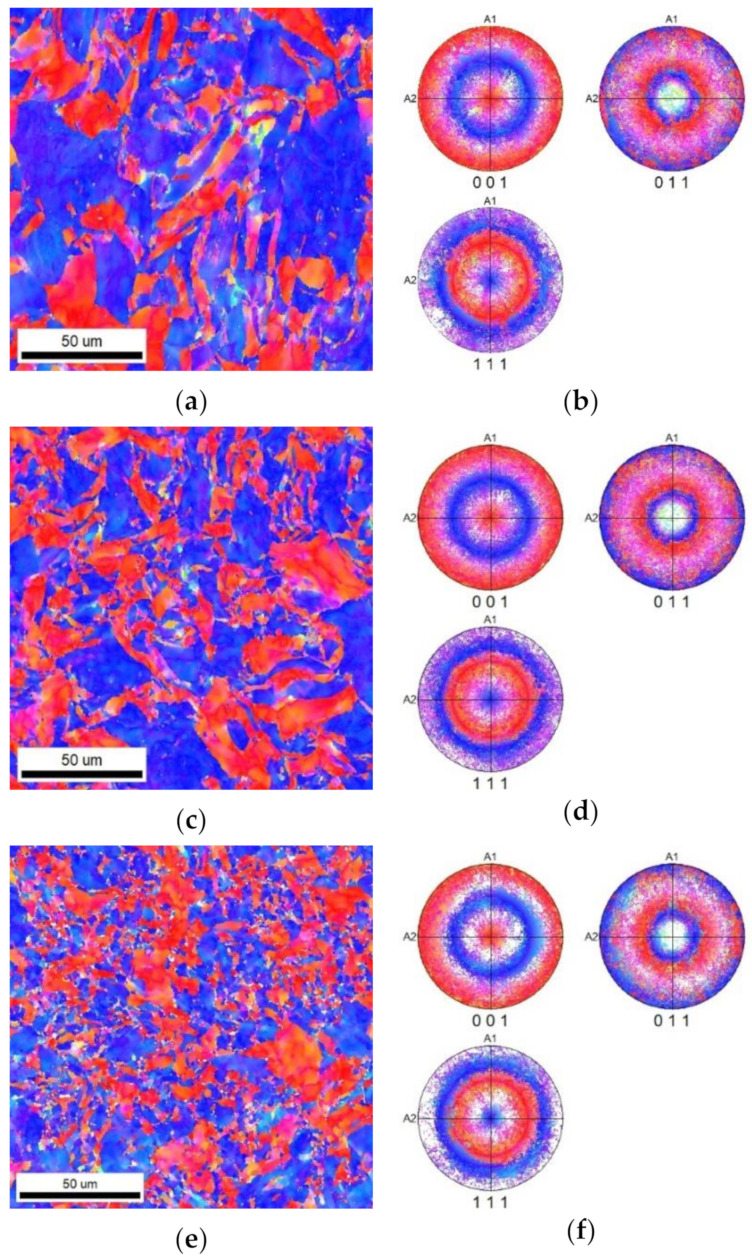
Orientation maps (OIMs) and pole figure (PF) images for swaged Cu (transversal): *bar20* (**a**,**b**), *bar15* (**c**,**d**), and *bar10* (**e**,**f**). The pole figures indicate the fiber texture of the material. The selection of orientations close to the (111) and (001) directions is visible from the orientation maps as well. The color code for the OIMs is given in Figure 1c.

The focus of structure observations was extended to examine the orientations of the individual grains in the cross-sectional cuts of the bars. Figure 3a displays the orientation image map (OIM) of the swaged structure of the *bar20* sample, revealing the formation of an intense fiber texture parallel to the swaging direction when swaged to the diameter of 20 mm. This texture is also evident in the corresponding pole figure (PF) images for the (001), (011), and (111) planes shown in Figure 3b.

The *bar15* sample also exhibited significant texture, as seen in Figure 3c,d, which present the OIM and PF images obtained from the transversal cut through the bar.

Finally, the OIM and PF images from the transversal cut through the *bar10* sample are shown in Figure 3e,f. As can be seen, this sample exhibited evident fiber texture as well. However, the grains in this sample were significantly refined compared to the *bar20* and *bar15* samples, which is in accordance with the grain size observations described in Section 3.1.

**Figure 4:**
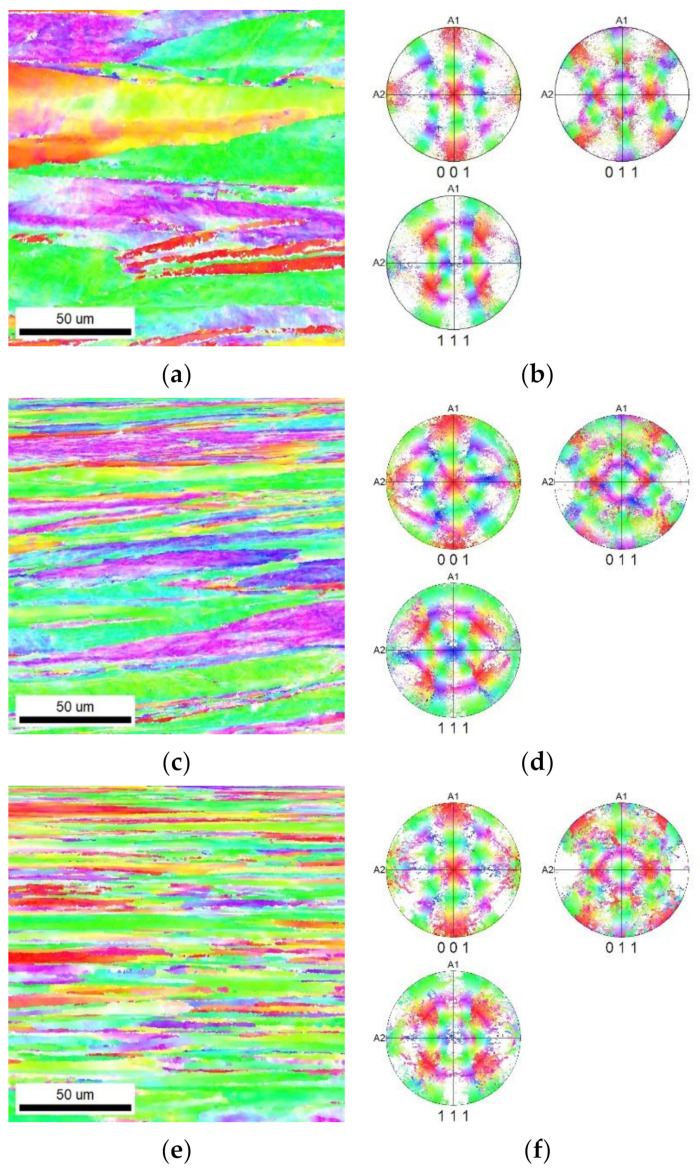
Orientation maps (OIMs) and polar figure (PF) images for swaged Cu samples (longitudinal): *bar20* (**a**,**b**), *bar15* (**c**,**d**), and *bar10* (**e**,**f**). Orientation maps clearly depict grains elongated in the horizontal direction, which is direction of axis of swaged bars. The color code for the OIMs is given in Figure 1c.

Figure 4a–c present the OIM images for the longitudinal cuts through the *bar20*, *bar15*, and *bar10* samples, respectively. These images demonstrate consistent texture orientations between swaging passes, showing no significant changes in this regard. However, noticeable differences were observed in terms of grain morphology; with a higher swaging ratio, the grains became visibly thinner and more elongated.

### 3.3. Electrical Conductivity

For the swaged conductors, the electrical conductivity values were measured using the method characterized above and then evaluated as a percentage of the IACS value (100% IACS is the value for the CP Cu). The results of the measurements conducted for all the investigated swaged bars are presented in Table 2. As is evident, the conductivity gradually increased with the decreasing conductor diameter, corresponding to an increasing reduction ratio.

### 3.4. Substructure

**Figure 5:**
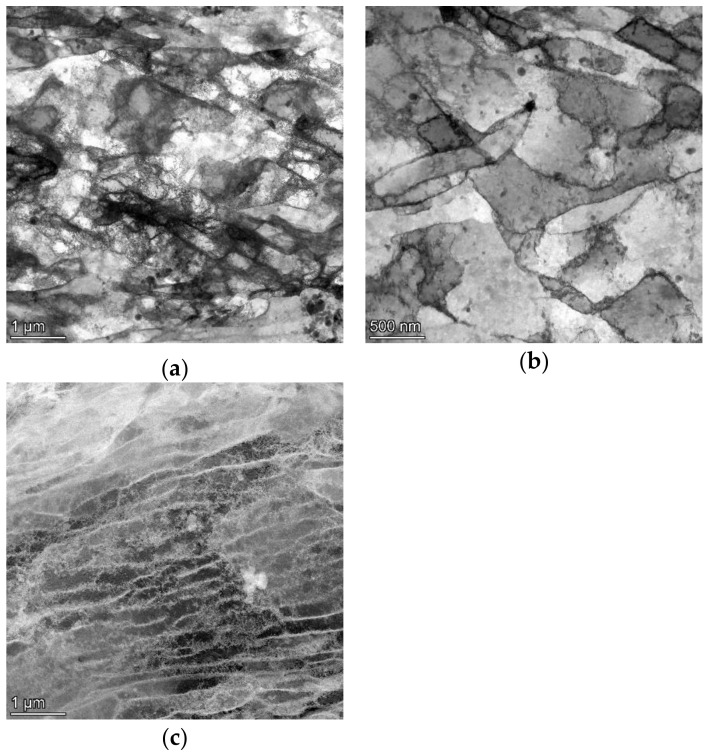
TEM substructure images for the *bar20* transversal cut (**a**), *bar15* transversal cut (**b**), and *bar15* longitudinal cut (**c**). We can see the development of the dislocation structure from areas with a higher dislocation density in *bar20* (**a**) to well-defined dislocation cells in *bar15* (**b**) and similar well-defined dislocated cells in the longitudinal direction in *bar10* (**c**).

Last but not least, TEM observations of the substructure were conducted. The analyses were carried out on foils from transversally cut samples for all the swaged bars, i.e., *bar20*, *bar15*, and *bar10*. Additional observations of foils acquired from the longitudinal cuts were performed when advantageous. The TEM image from the transversal cut from the *bar20* sample is depicted in Figure 5a. The bar evidently featured (relatively) large grains with a highly developed substructure and a high density of dislocations. The *bar15* sample clearly showed well-developed dislocation cells [58]. A scan acquired from the transversal cut from the *bar15* sample is depicted in Figure 5b, while Figure 5c depicts a scan from its longitudinal cut. As seen, the structure was restored during the swaging process; the structure scan from the transversal cut featured well-developed grains, and the presence of dislocations in the interiors of the grains was scarce. Nevertheless, the boundaries of the grains were evidently serrated, which can, in greater detail, be seen in the image acquired from the longitudinal cut depicted in Figure 5c. This phenomenon documents the accumulation of dislocations at grain boundaries within this sample.

## 4. Discussion

The results of the structure observations revealed that a gradual increase in the reduction ratios resulted in a gradual decrease in the grain size as regards the cross-section. Furthermore, with an increasing reduction ratio, the elongation of the grains became more pronounced. These trends were evident not only from the images depicting the transversal EBSD scans in Figure 3a,c,e but also from the EBSD images showing the longitudinal cuts in Figure 4a–c. The bimodality of the grain distribution decreased with the increasing reduction ratio, too; the *bar20* sample clearly exhibited the presence of fine grains together with grains with diameters of about 50 µm. However, the grain size range was not only smaller but also much more homogeneous in the *bar10* sample (Figure 2a–c). On the other hand, the preferential orientations of the grains did not show substantial changes during the swaging process. In other words, all the swaged bars exhibited the tendency to form the (111)‖SD (swaging direction) and (100)‖SD preferential texture fibers (Figure 3a–f). This behavior is typical for rotary swaged metals with the FCC lattice and is given by the character of the rotary swaging method, during which the axial material flow is dominant; such metals will generally exhibit the tendency to form the mentioned texture fibers [59,60]. Although the texture did not exhibit evident changes after the individual swaging passes, the imposed strain promoted evident changes in the substructure.

The grain refinement in the *bar20* sample was still limited due to the relatively low reduction ratio, as evident from the comparison of Figure 1a and Figure 3a. However, the imposed strain resulted in the accumulation of lattice defects, i.e., dislocations, and the development of a substructure. As a result, the rod swaged to the diameter of 20 mm exhibited relatively large grains with a well-developed substructure, characterized by a high density of dislocations and highly developed dislocation tangles and dislocation cells [58]. Further swaging to the diameter of 15 mm provided the swaged bar with additional deformation energy and led to structure restoration. In other words, the dislocation tangles and cells, which were evident in the structure of the *bar20* sample, transformed into dislocation walls within the *bar15* sample due to the supplied deformation energy [61]. Consequently, the transversal cut through the bar exhibited a structure with grains featuring only the minor presence of dislocations in their interiors. The fact that the dislocations were primarily accumulated in dislocation boundaries is documented by Figure 5c, which shows evidently serrated boundaries. Increasing the reduction ratio to the final value of 3.2, i.e., swaging the bar to the diameter of 10 mm, induced the most substantial (sub)structure changes. The grains were highly refined, as confirmed by all the SEM-EBSD analyses. Furthermore, the dislocations within the grains were restored.

Regarding the grain orientations, it is advantageous for a structure to have an optimized texture as it can facilitate electron movement during the transfer of electric current [62]. The presented results confirmed that all the swaged bars exhibited fiberlike textures, with grains elongated in the swaging direction corresponding to the direction of movement of electrons during the electric current transfer. This feature was most probably among the dominant factors contributing to the increased electrical conductivity observed for all the swaged bars (when compared to the original CP Cu, which featured large, substructure-free but equiaxed grains). The highest electrical conductivity of the *bar10* sample can thus be attributed to the combined effects of the favorable texture, i.e., elongation of the grains in the direction of electron movement.

Such behavior is generally unexpected but is a consequence of unusual processing. We can presume that conductivity in pure copper, where precipitates of other phases do not exist, is lowered mainly by grain boundaries. Conventionally annealed copper (600 °C/30 min) is homogeneous, with isotropic and equiaxed grains. Thus, we can expect a scattering event after 40 µm at least, as the grain size in a conventionally annealed sample is (21 ± 10) µm. Nevertheless, all rotary swaged samples had extremally prolonged grains along the swaging axis. The values we obtained from longitudinal cuts, shown in Figure 4, were twice or higher that of the conventionally annealed copper, despite the fact that we cannot see the whole length of the swaged grains because the maps’ areas are just 150 × 150 µm^2^. The conductivity was in any case enhanced as the probability of hitting the obstacle was significantly lowered. We can expect an increase in the conductivity with the increasing reduction in the swaged bars because of that, since grains became smaller on the cross-cut and longer in the longitudinal direction. The strongly deformed samples, namely, bar 15 and bar 10, showed higher levels of recovery and had lower amounts of dislocations in the volume of grains and thinner (sub)grain boundaries, see Figure 5; however, a detailed discussion of the various contributions to the scattering of conductivity electrons is beyond the scope of this article and will be investigated in future.

## 5. Conclusions

The presented study investigated the impact of the reduction ratio imposed during room-temperature rotary swaging on the (sub)structure and electrical conductivity of electroconductive copper bars. The key findings of our research can be summarized as follows:

(1) The increase in the reduction ratio resulted in a decrease in the sizes of grain cross-sections, accompanied by significant grain elongation in the swaging direction. These observations were consistent with the results obtained from the EBSD scans and TEM analyses.

(2) Rotary swaging induced substantial texture development in all swaged bars. The preferred texture fibers (111)‖SD and (100)‖SD were evident in the microstructure.

(3) The electrical conductivity of the swaged bars exhibited a gradual increase with successive swaging passes. Notably, the electrical conductivity of the copper bar swaged to a diameter of 10 mm reached 103.2% IACS, indicating an enhancement in electrical performance beyond the conductivity of the original CP Cu.

Overall, the favorable increase in electrical conductivity observed in swaged bars can be attributed to the synergistic effects of the advantageous texture, specifically the elongation of the grains in the direction of moving electrons and recovery of grain interiors within the swaging process. The combined outcomes of grain refinement, substructure development, and texture evolution highlight the significance of optimized deformation processing, such as rotary swaging, as a promising approach to enhancing the performance of copper while maintaining a high electrical conductivity.

## Figures and Tables

**Table 1:** Reduction ratios for swaged electroconductive bars with sample denotations.

Bar Diameter [mm]	20	15	10
Reduction ratio	1.8	2.4	3.2
Sample denotation	*bar20*	*bar15*	*bar10*

**Table 2:** Electric conductivity for swaged conductors.

Sample	CP Cu	20	15	10
Electric conductivity (% IACS)	100	101.9	102.2	103.2

## Data Availability

The original data supporting the research are not publicly available, but the portion of data that is not confidential is available on request from the corresponding author.
